# Infertility and pregnancy outcomes among adults with primary ciliary dyskinesia

**DOI:** 10.1093/hropen/hoae039

**Published:** 2024-06-18

**Authors:** Leonie D Schreck, Eva S L Pedersen, Katie Dexter, Michele Manion, Living with PCD Study Advisory Group, Sara Bellu, Isabelle Cizeau, Katie Dexter, Lucy Dixon, Trini López Fernández, Susanne Grieder, Catherine Kruljac, Michele Manion, Bernhard Rindlisbacher, Hansruedi Silberschmidt, Emilie Wattellier, Nathalie Massin, Bernard Maitre, Myrofora Goutaki, Claudia E Kuehni

**Affiliations:** Institute of Social and Preventive Medicine, University of Bern, Bern, Switzerland; Graduate School for Health Sciences, University of Bern, Bern, Switzerland; Institute of Social and Preventive Medicine, University of Bern, Bern, Switzerland; PCD Support UK, Buckingham, UK; PCD Foundation, Minneapolis, MN, USA; IVF Center, American Hospital of Paris, Neuilly-sur-Seine, France; Institut National de la Santé et de la Recherche Médicale (INSERM), IMRB, Université Paris-Est Créteil, Créteil, France; Department of Pneumology, Centre Hospitalier Intercommunal de Créteil, Créteil, France; Institute of Social and Preventive Medicine, University of Bern, Bern, Switzerland; Division of Paediatric Respiratory Medicine and Allergology, Department of Paediatrics, Inselspital, University Hospital, University of Bern, Bern, Switzerland; Institute of Social and Preventive Medicine, University of Bern, Bern, Switzerland; Division of Paediatric Respiratory Medicine and Allergology, Department of Paediatrics, Inselspital, University Hospital, University of Bern, Bern, Switzerland

**Keywords:** primary ciliary dyskinesia, infertility, ectopic pregnancy, assisted reproduction, epidemiology, orphan disease, genotype

## Abstract

**STUDY QUESTION:**

What is the prevalence of infertility and ectopic pregnancies among individuals with primary ciliary dyskinesia (PCD)?

**SUMMARY ANSWER:**

We found that 39 of 50 men (78%) and 72 of 118 women (61%) with PCD were infertile and that women with PCD had an increased risk of ectopic pregnancies (7.6 per 100 pregnancies, 95% CI 4.7–12.2).

**WHAT IS KNOWN ALREADY:**

PCD is a heterogeneous multiorgan disease caused by mutations in genes required for the function and structure of motile cilia. Previous studies identified a link between PCD and infertility, but original data on prevalence of infertility and risk of ectopic pregnancies, the use and efficacy of medically assisted reproduction (MAR), and the association of fertility with PCD genotype are extremely limited.

**STUDY DESIGN, SIZE, DURATION:**

We performed a cross-sectional survey about fertility within the *Living with PCD* study (formerly COVID-PCD). *Living with PCD* is an international, online, participatory study that collects information directly from people with PCD. People with PCD of any age from anywhere in the world can participate in the study. At the time of the survey, 482 adults with PCD were registered within the *Living with PCD* study.

**PARTICIPANTS/MATERIALS, SETTING, METHODS:**

We sent a questionnaire on fertility on 12 July 2022, to all participants older than 18 years enrolled in the *Living with PCD* study. Responses were collected until 8 March 2023. The fertility questionnaire covered topics related to pregnancy attempts, use of MAR, and pregnancy outcomes. Data were collected via the Research Electronic Data Capture (REDCap) platform. We defined infertility as failure to achieve a clinical pregnancy after 12 months or use of MAR for at least one pregnancy.

**MAIN RESULTS AND THE ROLE OF CHANCE:**

In total, 265 of 482 adult participants (55%) completed the fertility questionnaire. Among 168 adults who had tried to conceive, 39 of 50 men (78%) and 72 of 118 women (61%) were infertile. Of the infertile men, 28 had tried MAR, and 17 of them (61%) fathered a child with the help of MAR. Among infertile women, 59 had used MAR, and 41 of them (69%) became pregnant with the help of MAR. In our population, women with PCD showed a relatively high risk of ectopic pregnancies: 1 in 10 women who became pregnant had at least one ectopic pregnancy and 7.6% of pregnancies were ectopic (95% CI 4.7–12.2). We evaluated the association between fertility and affected PCD genes in 46 individuals (11 men, 35 women) with available genetic and fertility information, and found differences between genotypes, e.g. all five women with a mutation in *CCDC40* were infertile and all five with *DNAH11* were fertile.

**LIMITATIONS, REASONS FOR CAUTION:**

The study has limitations, including potential selection bias as people experiencing problems with fertility might be more likely to fill in the questionnaire, which may have influenced our prevalence estimates. We were unable to validate clinical data obtained from participant self-reports owing to the anonymous study design, which is likely to lead to recall bias.

**WIDER IMPLICATIONS OF THE FINDINGS:**

The study underlines the need for addressing infertility in routine PCD care, with a focus on informing individuals with PCD about their increased risk. It emphasizes the utility and efficacy of MAR in PCD-related infertility. Additionally, women attempting conception should be made aware of the increased risk of ectopic pregnancies and seek systematic early consultation to confirm an intrauterine pregnancy. Fertility, efficacy of MAR, and risk for adverse pregnancy outcomes differ between people with PCD—depending on genotypes—and close monitoring and support might be needed from fertility specialists to increase chances of successful conception.

**STUDY FUNDING/COMPETING INTEREST(S):**

Our research was funded by the Swiss National Science Foundation, Switzerland (SNSF 320030B_192804), the Swiss Lung Association, Switzerland (2021-08_Pedersen), and we also received support from the PCD Foundation, USA; the Verein Kartagener Syndrom und Primäre Ciliäre Dyskinesie, Germany; the PCD Support UK, UK; and PCD Australia, Australia. M. Goutaki received funding from the Swiss National Science Foundation, Switzerland (PZ00P3_185923). B. Maitre participates in the RaDiCo-DCP funded by INSERM France. The study authors participate in the BEAT-PCD Clinical Research Collaboration supported by the European Respiratory Society. All authors declare no conflict of interest.

**TRIAL REGISTRATION NUMBER:**

ClinicalTrials.gov ID NCT04602481.

WHAT DOES THIS MEAN FOR PATIENTS? Primary ciliary dyskinesia (PCD) is a rare genetic disease. People with PCD can have issues conceiving. It is unclear how many struggle to have children, and how many are able to have children with medical help. We also do not know if women with PCD tend to have more ectopic pregnancies (pregnancies outside of the uterus) than the general population. How did we answer these questions? We sent a questionnaire about fertility to all participants in the *Living with PCD* study. The *Living with PCD* study is an online study. It collects information directly from people with PCD from all over the world. How many people with PCD struggled to have children? Eight out of 10 men and 6 out of 10 women experienced problems conceiving. How many were successful with help? Among those who struggled, two out of three were able to have a child with the help of fertility treatments. Did women with PCD have more ectopic pregnancies? Women with PCD had more ectopic pregnancies than the general population. We found 7 out of 100 pregnancies in PCD were ectopic, compared to only two in 100 pregnancies in the general population. We believe that people with fertility problems were more likely to complete our questionnaire, which means that the true risk of ectopic pregnancy in PCD may be lower than we found in our study. But ectopic pregnancies can lead to serious complications. Thus, the authors of this paper think that fertility specialists should inform women with PCD about their increased risk. We also believe that women with PCD should see their gynaecologist early in their pregnancy to confirm that the pregnancy is inside the uterus. The authors suggest addressing fertility problems in routine PCD care, with the help of fertility specialists with an interest in PCD.

## Introduction

Primary ciliary dyskinesia (PCD) can lead to infertility and adverse pregnancy outcomes. PCD is a rare heterogeneous multiorgan disease with an estimated prevalence of around 1 in 7500 (Hannah *et al.*, 2022). It is caused by mutations in genes needed for the function and structure of motile cilia. More than 50 disease-causing genes have been described leading to varying degrees of PCD symptoms (Lucas *et al.*, 2020; Wheway *et al.*, 2021). Defects in motile cilia lining the respiratory tract contribute to chronic airway disease, a common characteristic in patients with PCD. Furthermore, motile cilia play essential roles in the reproductive systems of men and women. In men with PCD, mutations in motile cilia in the efferent ductules may lead to oligozoospermia (reduced sperm count) or azoospermia (no sperm) due to obstructive sperm stasis (Aprea *et al.*, 2021). Sperm flagella share similar axonemal structures with motile cilia, with mutations leading to asthenozoospermia (sperm dysmotility) or teratozoospermia (morphological abnormalities of sperm) (Sironen *et al.*, 2020). In women, reduced function of motile cilia lining the fallopian tubes and the uterus may be the cause of reduced fertility (Marchini *et al.*, 1992; Lyons *et al.*, 2006; Raidt *et al.*, 2015; Pearson-Farr *et al.*, 2023), possibly leading to impaired oocyte transport and an increased risk of ectopic pregnancies (Lin *et al.*, 1998; McLean and Claman, 2000; Blyth and Wellesley, 2008).

Counselling patients with PCD about their likelihood of conceiving is difficult and based on limited available data. We lack information about the extent of fertility issues and ectopic pregnancies, and the use and success rates of medically assisted reproduction (MAR). Additionally, fertility is suggested to vary depending on the PCD gene affected but little is known about the exact associations. Fertility is not regularly assessed in all people with PCD and this information is not routinely recorded in medical records or existing registries and clinical datasets (Werner *et al.*, 2016; Goutaki *et al.*, 2017, 2019; Ardura-Garcia *et al.*, 2020). Evidence comes almost exclusively from case reports and case series. In a systematic review and meta-analysis from 2016, estimates of infertility prevalence were very heterogenous among seven studies ranging from 15% to 79% (Goutaki *et al.*, 2016). A recent narrative review confirmed the low level of evidence (Newman *et al.*, 2023). The largest study on infertility to date from 2017 included 85 individuals from PCD hospitals in France and Belgium (Vanaken *et al.*, 2017); 61% of women and 76% of men were considered infertile, and risk of infertility differed by involved PCD gene. In this study, 6 of 22 women and 15 of 37 men had benefitted from the use of MAR. Success of MAR has also been described in case reports—particularly IVF for women and ICSI for men (Jayasena and Sironen, 2021; Newman *et al.*, 2023). Data on the frequency of ectopic pregnancies are scarce with only four ectopic pregnancies reported in case reports so far (Lin *et al.*, 1998; McLean and Claman, 2000; Blyth and Wellesley, 2008; Newman *et al.*, 2023). It is impossible to extrapolate the prevalence of ectopic pregnancies from these case reports.

In the largest study on fertility in PCD to date, we studied fertility and pregnancy outcomes of adults with PCD, including the prevalence of infertility, use and efficacy of MAR, the prevalence of ectopic pregnancies, and the association of fertility with PCD genotype.

## Materials and methods

### Study design, study population, and ethics

We used data from a cross-sectional questionnaire about fertility conducted as part of the *Living with PCD* study (formerly COVID-PCD). *Living with PCD* is an international, online, participatory study that collects information directly from people with PCD. A detailed study protocol has been published (Pedersen *et al.*, 2021). In short, *Living with PCD* was set up during the coronavirus disease 2019 (COVID-19) pandemic in 2020 at the University of Bern, Switzerland, in collaboration with PCD support groups worldwide. The study continuously monitored people with PCD during the pandemic and we have now started examining other aspects relevant to this population, such as fertility. We identified this topic as of great interest to individuals living with PCD themselves based on the feedback from participants directly. Questionnaires are available in five languages (English, French, German, Italian, and Spanish) and people with PCD of any age from anywhere in the world can participate in the study. The study is advertised and distributed via PCD patient support groups worldwide and people with PCD or their caregivers, such as parents, actively contribute to study design. A study advisory group consisting of support group representatives is involved in all relevant decisions regarding the study. The study is anonymous, with participants or their caregivers providing informed consent upon study enrolment. Participants retain the right to withdraw from the study at any time by contacting the study team. The cantonal ethics committee of Bern, Switzerland, approved the study (study ID: 2020-00830).

### Study procedure

Participants self-registered via the study website (www.pcd.ispm.ch) and received a baseline questionnaire which included questions on demographic characteristics, diagnostic information including genetic results and symptoms, followed by shorter weekly follow-up questionnaires. We sent a special questionnaire dedicated to fertility on 12 July 2022, to all participants older than 18 years enrolled in the *Living with PCD* study. Non-responders received up to three reminders via email.

We collected responses until 8 March 2023. Participants entered data directly into an online database using the Research Electronic Data Capture (REDCap) platform (Harris *et al.*, 2009), which is hosted by the Swiss medical registries and data linkage centre at the University of Bern, Switzerland.

### Fertility questionnaire

We developed the fertility questionnaire in close collaboration with fertility specialists, reproductive endocrinologists, and participants of the study advisory group where participants made suggestions to improve understanding of the questions. The questions and answer categories are listed in Supplementary Table S1. Members of the research team translated the questionnaires and two native-speaking members of the team or from the study advisory group checked translations independently.

### Information on fertility and pregnancy

**Figure 1.**
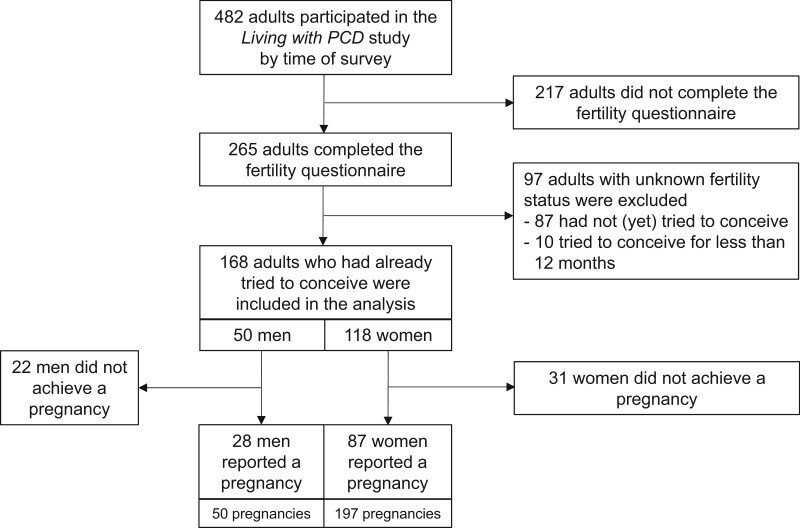
Flowchart of adults with primary ciliary dyskinesia in Living with PCD* who were invited and completed the fertility questionnaire (N = 482). *Living with PCD is an international, online, participatory study that collects information by questionnaire directly from people with PCD. PCD, primary ciliary dyskinesia.

We asked participants if they had ever tried to conceive a child; if so, if they had used MAR, and if the attempt resulted in a pregnancy. We included all participants with available fertility data in our analyses and thus excluded participants who had never tried to conceive or had tried for <12 months (Fig. 1). We categorized people as fertile if they achieved pregnancy naturally without MAR (Zegers-Hochschild *et al.*, 2017). Consequently, we defined them as infertile if they failed to achieve a clinical pregnancy after 12 months or if they used MAR for at least one pregnancy (Zegers-Hochschild *et al.*, 2017; World Health Organization, 2019). We subclassified infertile individuals according to the use of MAR as ‘pregnancy with MAR’, ‘no pregnancy despite MAR’, ‘no pregnancy, no MAR’. We asked all participants about fertility tests and results, as well as other factors that may contribute to infertility, such as abnormal levels of fertility hormones or diseases of the reproductive system (Vander Borght and Wyns, 2018). The definition of these factors can be found in Supplementary Table S2.

Among participants reporting a pregnancy, we asked about the mode of fertilization and the outcome of the pregnancy. We grouped pregnancy outcome as live birth, pregnancy loss/stillbirth, ectopic pregnancy, abortion, or ongoing pregnancy. To assess how fertility differed by genotype, we grouped reported PCD genes into categories based on associated defects: genes involved in the coding of proteins for dynein structure, dynein assembly, microtubular stabilization/nexin–dynein regulatory complex, radial spoke and central complex, and other function (Shoemark *et al.*, 2021). We excluded participants who reported two heterozygous variants in different genes.

### Data analyses

We assessed the prevalence of infertility and fertility outcomes stratified by sex. We described use of MAR and examined the efficacy of MAR by determining the individuals who achieved pregnancy with MAR among all who underwent MAR. For women, we calculated the prevalence of ectopic pregnancies as the proportion of ectopic pregnancies among all reported pregnancies and all women trying to conceive. Since not all *Living with PCD* participants completed the fertility questionnaire, to test the robustness of our findings, we also calculated the resulting minimum prevalence of ectopic pregnancies for our entire study population. We hypothesized that women with an ectopic pregnancy might have been more likely to fill in the questionnaire and assumed that women who did not complete the questionnaire had no ectopic pregnancy. For simplicity, we assumed that all women in *Living with PCD* had the same median number of pregnancies, regardless of questionnaire completion. Based on these two assumptions, we calculated the minimum prevalence as the proportion of reported ectopic pregnancies out of all hypothetical pregnancies and of all women trying to conceive in the *Living with PCD* study. Finally, we assessed fertility status by reported PCD genotype. We calculated Wilson 95% CIs for proportions.

## Results

### Characteristics of the study participants

At the time of the study, of 482 adults with PCD who had registered within the *Living with PCD* study, 265 of them (55%) completed the fertility questionnaire (Fig. 1, Table 1). Most of them were women (180; 68%) with a median age of 44 years (IQR 33–54) at survey. The respondents came from 33 countries with the largest number from the USA (41; 15%), England (39; 15%), Germany (37; 14%), and Switzerland (26; 10%). Compared with non-responders, people who completed the fertility questionnaire were older (Supplementary Table S3).

**Table 1.** Characteristics of adults with primary ciliary dyskinesia participating in Living with PCD* who completed the fertility questionnaire, overall and by sex (N = 265).

		Overall	Men	Women
		n (%)	n (%)	n (%)
		N = 265	N = 85	N = 180
**Age at survey (years)**	Median (IQR; range)	44 (33–54; 19–87)	48 (34–56; 19–77)	43 (33–52; 19–87)
	18–30 y	47 (18)	12 (14)	35 (19)
	31–45 y	100 (38)	28 (33)	72 (40)
	> 45 y	118 (45)	45 (53)	73 (41)
**Fertility information**	Tried to conceive	168 (63)	50 (59)	118 (66)
	Not (yet) tried to conceive	87 (33)	30 (35)	57 (32)
	Tried to conceive for <12 months	10 (4)	5 (6)	5 (3)
	**Available fertility status**	**n = 168**	**n = 50**	**n = 118**
	Fertile	57 (34)	11 (22)	46 (39)
	Infertile	111 (66)	39 (78)	72 (61)
	Achieved pregnancy	115 (68)	28 (56)	87 (74)
	Did not achieve pregnancy	53 (32)	22 (44)	31 (26)
**Countries** ^a^	USA	41 (15)	12 (14)	29 (16)
	England	39 (15)	11 (13)	28 (16)
	Germany	37 (14)	9 (11)	28 (16)
	Switzerland	26 (10)	9 (11)	17 (9)
	Italy	19 (7)	8 (9)	11 (6)
	France	16 (6)	6 (7)	10 (6)
	Australia	12 (5)	4 (5)	8 (4)
	Canada	10 (4)	1 (1)	9 (5)
	Netherlands	9 (3)	4 (5)	5 (3)
	Scotland	7 (3)	3 (4)	4 (2)
	Norway	6 (2)	3 (4)	3 (2)
	Denmark	5 (2)	2 (2)	3 (2)
	other European countries	30 (11)	12 (14)	18 (10)
	other countries	8 (3)	1 (1)	7 (4)

All characteristics are presented as n and column % unless otherwise stated. IQR, interquartile range. PCD, primary ciliary dyskinesia. y, years.

* *Living with PCD* is an international, online, participatory study that collects information by questionnaire directly from people with PCD.

Countries with N ≥ 5 displayed in table, countries with N < 5 were categorized into other European countries and other countries. Other European countries include Austria, Belgium, Croatia, Cyprus, Czech Republic, Hungary, Ireland, Jersey, Northern Ireland, Poland, Portugal, Spain, Sweden, and Wales. Other countries include Brazil, Cameroon, Hong Kong, Israel, Panama, Puerto Rico, and South Africa.

### Fertility and pregnancy outcomes of men

**Figure 2.**
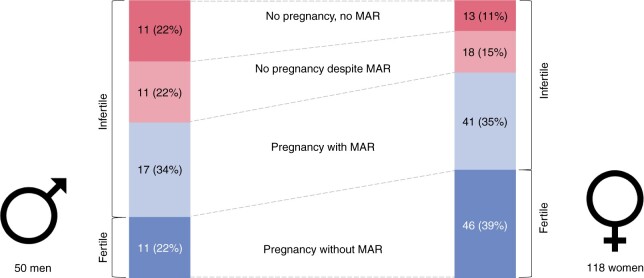
Fertility status of adults participating in Living with PCD* who completed the fertility questionnaire and have ever tried to conceive, by sex (N = 168). *Living with PCD is an international, online, participatory study that collects information by questionnaire directly from people with PCD. MAR, medically assisted reproduction. PCD, primary ciliary dyskinesia.

Among 50 men who had tried to conceive, 11 men (22%) were fertile, and 39 (78%) were infertile (Fig. 2, Table 1). Of the infertile men, 28 had tried MAR, and 17 of them (61%) fathered a child with the help of MAR. Overall, 28 men (56%) fathered a child with or without the help of MAR. The men in our study were a median of 31 years old (IQR 28–35) when they first tried to conceive a child. Among 62 men who reported sperm analysis, only 3 (5%) had a normal test result (Supplementary Table S4).

Overall, the 28 men who conceived reported a total of 50 pregnancies. In more than half of those pregnancies (28; 56%), they had used MAR. Types of fertilization most commonly used for successful pregnancies were ICSI (15; 41%) and IVF (9; 24%). Most of the pregnancies resulted in live births (39; 78%), but there were also 10 pregnancy losses or stillbirths (20%) reported by seven men (Table 2).

**Table 2.** Mode of fertilization and pregnancy outcomes for pregnancies reported by adults in Living with PCD* who completed the fertility questionnaire, by sex (N = 247).

	All pregnancies reported by men	All pregnancies reported by women
	n (%)	n (%)
	N = 50	N = 197
**Mode of fertilization**		
Pregnancy without MAR	22 (44)	125 (63)
Pregnancy with MAR	28 (56)	70 (36)
Unknown mode of fertilization	0 (0)	2 (1)
Types of MAR used^a^	n = 37	n = 113
Ovarian stimulation	0 (0)	26 (23)
Artificial insemination	2 (5)	5 (4)
IVF	9 (24)	55 (49)
ICSI	15 (41)	20 (18)
Sperm donation	4 (11)	4 (4)
Oocyte donation	0 (0)	1 (1)
Partner used MAR	7 (19)	1 (1)
Unknown type of MAR	0 (0)	1 (1)
**Pregnancy outcomes**		
Live birth	39 (78)^b^	124 (63)^c^
Pregnancy loss/stillbirth	10 (20)	44 (22)
Ectopic pregnancy	0 (0)	15 (8)
Abortion	1 (2)	11 (6)
Ongoing pregnancy	0 (0)	3 (2)^d^

All characteristics are presented as n and column %. MAR, medically assisted reproduction. PCD, primary ciliary dyskinesia.

* *Living with PCD* is an international, online, participatory study that collects information by questionnaire directly from people with PCD.

Types of MAR exceed the number of pregnancies with MAR as more than one type of MAR was reported for each pregnancy on average.

Four live births were the result of sperm donations.

Five women reported the birth of twins (= 129 babies born).

One ongoing pregnancy was the result of an oocyte donation.

### Fertility and pregnancy outcomes of women

Among the 118 women who reported pregnancy attempts, 46 (39%) became pregnant naturally, and 72 women (61%) experienced infertility (Fig. 2, Table 1). Among infertile women, 59 had used MAR, and 41 of them (69%) became pregnant with the help of MAR. A total of 87 women (74%) became pregnant at least once. The women in our study were a median of 30 years old (IQR 27–32) when they first tried to conceive a child (Supplementary Table S5).

Overall, 87 women who managed to become pregnant reported a total of 197 pregnancies (Table 2). Two-thirds (125; 63%) of the pregnancies were conceived without MAR. For successful pregnancies, IVF (55; 49%), ovarian stimulation with or without IUI (26; 23%) or ICSI (20; 18%) were the most commonly used types of MAR. The 197 pregnancies resulted in 124 (63%) live births, 44 (22%) miscarriages, 15 (8%) ectopic pregnancies, and 11 (6%) abortions. Three (2%) pregnancies were ongoing at the time of the survey. Overall, 1 in 10 women who became pregnant had at least one ectopic pregnancy.

We estimated the prevalence of ectopic pregnancies among women to be 7.6 per 100 pregnancies (95% CI 4.7–12.2) and 12.7 per 100 women trying to conceive (95% CI 7.9–19.9) (Table 3). Assuming that there were no other ectopic pregnancies among women who did not complete the questionnaire and that the number of pregnancies per woman was the same in the whole population, we calculated a minimum prevalence of ectopic pregnancies for the whole *Living with PCD* cohort of 4.2 per 100 pregnancies (95% CI 2.6–6.9) and 7.1 per 100 women trying to conceive (95% CI 4.4–11.4).

**Table 3.** Frequency of ectopic pregnancies among all women participating in Living with PCD* who completed the fertility questionnaire and among all women in *Living with PCD*.

Ectopic pregnancies	Women in *Living with PCD* who completed fertility questionnaire	**Minimum prevalence: all women in *Living with PCD*** ^a^
**- Per 100 pregnancies**	7.6 (95% CI 4.7–12.2)	4.2 (95% CI 2.6–6.9)
**- Per 100 women trying to conceive**	12.7 (95% CI 7.9–19.9)	7.1 (95% CI 4.4–11.4)

PCD, primary ciliary dyskinesia.

* *Living with PCD* is an international, online, participatory study that collects information by questionnaire directly from people with PCD.

We calculated Wilson 95% CIs for proportions. We based the calculation of the minimum prevalence on two assumptions: women who did not complete the questionnaire had no ectopic pregnancy and all women in *Living with PCD* had the same median number of pregnancies. Of 180 women who completed the survey, 118 tried to conceive and reported 197 pregnancies. Of 142 women who did not complete the survey, 93 additional women would have tried to conceive and would have 156 additional pregnancies. Overall, we would count 211 women trying to conceive and 353 pregnancies among all women in *Living with PCD*. 15 ectopic pregnancies per 353 pregnancies of all women in *Living with PCD *=* *4.2 ectopic pregnancies per 100 pregnancies. 15 ectopic pregnancies per 211 women trying to conceive in *Living with PCD *=* *7.1 ectopic pregnancies per 100 women trying to conceive.

Most of the nine women who reported ectopic pregnancies did not report any risk factors for their first ectopic pregnancy other than PCD, such as MAR use, or previous tubal surgery (Supplementary Table S6). For six women, the ectopic pregnancy was their first pregnancy. Five women reported more than one ectopic pregnancy and seven women reported one or more live births before or after the ectopic pregnancy.

### Association of fertility status with genotype

We assessed differences in fertility between genotypes for 46 people (11 men, 35 women) with available genetic and fertility information (Table 4). Infertility was reported in all individuals in the dynein assembly group [*DNAAF3* n = 1, *ZMYND10* n = 2, *PIH1D3* (*DNAAF6*) n = 1], the microtubular stabilization/nexin–dynein regulatory complex group [*CCDC39* n = 1, *CCDC40* n = 6, *CCDC65* (*DRC2*) n = 1], and the group with mutations in radial spoke and central complex (*RSPH9* n = 3, *HYDIN* n = 1, *RSPH1* n = 1). All five women with a mutation in *DNAH11* reported to be fertile. The pattern was more heterogeneous for other genes. Among women with a mutation in *DNAH5*, eight reported being fertile and seven reported being infertile.

**Table 4.** Gene groups and single PCD genes of men and women participating in Living with PCD* with available genetic and fertility information who completed the fertility questionnaire, by fertility status (N = 46).

**Gene groups** ^a^	PCD gene		Men		Women	
		Total	Fertile	Infertile	Fertile	Infertile
		N = 46	n = 3	n = 8	n = 16	n = 19
Dynein structure	*DNAH5*	17	2	–	8	7
	*DNAH11*	5	–	–	5	–
	*DNAI1*	4	–	1	3	–
	*DNAH9*	1	–	1	–	–
	*ODAD1* (*CCDC114*)	1	–	1	–	–
Dynein assembly	*DNAAF3*	1	–	–	–	1
	*ZMYND10*	2	–	–	–	2
	*PIH1D3* (*DNAAF6*)	1	–	1	–	–
Microtubular stabilization/nexin–dynein regulatory complex	*CCDC39*	1	–	1	–	–
	*CCDC40*	6	–	1	–	5
	*CCDC65* (*DRC2*)	1	–	1	–	–
Radial spoke and central complex	*RSPH9*	3	–	–	–	3
	*HYDIN*	1	–	–	–	1
	*RSPH1*	1	–	1	–	–
Other function	*RPGR*	1	1	–	–	–

PCD, primary ciliary dyskinesia.

* *Living with PCD* is an international, online, participatory study that collects information by questionnaire directly from people with PCD.

We grouped affected genes according to the corresponding ultrastructural defect, as done previously (Shoemark *et al.*, 2021).

## Discussion

In the largest study on fertility in PCD to date, we showed a high prevalence of infertility among people with PCD, with two-thirds of women and four-fifths of men reporting difficulties conceiving. Notably, two-thirds of infertile individuals who underwent MAR successfully conceived. We counted 15 ectopic pregnancies in our study, which corresponds to a prevalence of ectopic pregnancies of around 8 ectopic pregnancies per 100 pregnancies.

### Strengths and limitations

For the first time in PCD, we collected data on fertility status from adults with PCD worldwide. By directly asking people with PCD, we not only collected information on live births but also on other birth outcomes that are often not well recorded in medical charts to ensure accurate prevalence estimates. However, we were not able to validate clinical data obtained from participant self-reports owing to the anonymous study design, which is likely to lead to recall bias. For example, we cannot exclude that miscarriages of unknown localization have been incorrectly described by patients as ectopic pregnancies in some instances. People struggling with fertility might have been more likely to complete the fertility questionnaire, which could have led to selection bias and influenced our prevalence estimates. Calculating the minimum prevalence of ectopic pregnancies for the entire study population confirmed the robustness of our findings. However, it is still possible that women who did not complete the questionnaire had more pregnancies. Owing to the complexity of fertility status, which depends on various factors, we cannot causally link infertility to PCD in our study population. Other reasons for infertility include partner’s fertility problems, infrequent sexual intercourse, sexually transmitted diseases, or other urological or gynaecological conditions, some of which we have assessed in our questionnaire (Supplementary Tables S4 and S5). Without a clinical assessment, we cannot determine the definite reason for infertility.

### Comparison with previous research and general population

Our findings quantified the high risk of infertility among individuals with PCD, confirming differences of fertility status between men and women. Our infertility prevalence estimates were higher (66% overall) than those estimated in the systematic review and meta-analysis from 2016 where infertility in seven studies was ranging from 15% to 79% with a weighted mean of 30% (Goutaki *et al.*, 2016). In three studies with results stratified by sex, the authors estimated male infertility at 100%, but most studies included only a few male adults. In our study, 22% of the men fathered a child without the help of MAR. Our results are in line with results from the largest study on infertility to date by Vanaken from 2017. They assessed fertility in a cohort of patients recruited from PCD hospitals in France and Belgium and estimated the risk of infertility among 36 women to be 61% and among 49 men to be 76% (Vanaken *et al.*, 2017). In a narrative review published in 2023, prevalence was not estimated as the quality of evidence from case reports was not considered good enough (Newman *et al.*, 2023).

We showed an increased risk of ectopic pregnancies among women with PCD compared with the general population. Limited data on ectopic pregnancies among women in the general population of high-income countries estimate the risk to be at maximum two ectopic pregnancies per 100 pregnancies (Van Den Eeden *et al.*, 2005; Jurkovic and Wilkinson, 2011; Panelli *et al.*, 2015). Our results suggest that in women with PCD, this risk is 3.8-fold (95% CI 2.4–6.1) increased. To date, data on ectopic pregnancies among women with PCD are controversial. Both spontaneous pregnancies and infertility have been reported for women with dyskinetic ciliary activity (Bleau *et al.*, 1978; McComb *et al.*, 1986; Lurie *et al.*, 1989; Marchini *et al.*, 1992; Raidt *et al.*, 2015). Even though pathophysiological considerations suggest an increased risk of ectopic pregnancy, the involvement of cilia in the transport of the oocyte in the uterine tractus and, consequently, female infertility is not well understood. Only four reports of ectopic pregnancies among women with PCD existed before this study (Lin *et al.*, 1998; McLean and Claman, 2000; Blyth and Wellesley, 2008; Newman *et al.*, 2023) with Vanaken not reporting any ectopic pregnancies among their 36 women (Vanaken *et al.*, 2017). We will need large clinical studies to confirm our results.

We provided new insights into the associations of fertility with genotype. In line with previous research, we showed that people with mutations in *CCDC40* are more likely to be infertile (Vanaken *et al.*, 2017; Liu *et al.*, 2021). In contrast, *DNAH5* has not previously been associated with infertility in women (Newman *et al.*, 2023), but 7 out of 15 women in our study with a mutation in *DNAH5* were infertile. Conversely, for *DNAH11*, we reported only fertile individuals, whereas previous publications showed both fertile and infertile individuals (Brennan *et al.*, 2021; Newman *et al.*, 2023). We have also confirmed the importance of assessing other factors that affect fertility when interpreting the results. For example, a man with a *ODAD1* (*CCDC114)* mutation, which is not associated with a higher risk of infertility (Kos *et al.*, 2022), was found to be infertile, possibly due to another severe condition he reported.

### Interpretation and implication for future research

Fertility is a topic that needs to be addressed in regular PCD care and people with PCD need to be informed about their possible increased risk of infertility (Schreck *et al.*, 2024). However, not every person with PCD is infertile, which needs to be communicated as well. Additionally, we underlined the usefulness and effectiveness of MAR in PCD and suggest that it should be offered to infertile patients with PCD early on. For people with PCD who do not wish to get pregnant, adequate birth control should be in place. Women who are trying to conceive should be made aware of the potentially increased risk of ectopic pregnancy so that they react early and consult their gynaecologist to confirm the intrauterine position of the pregnancy before symptoms arise. It is important to note that the risk of ectopic pregnancy in our population was not related to the use of MAR. Future studies will need to assess infertility among people with PCD in even larger international clinical cohorts that link hospital data with fertility information and study the efficacy of MAR in more detail.

## Conclusion

We quantified a high risk of infertility among people with PCD. MAR was often used successfully in PCD-related infertility. Women attempting conception should be aware of the potentially elevated risk of ectopic pregnancies and seek systematic early consultation to confirm intrauterine pregnancy (4–5 weeks of gestation). Fertility, efficacy of MAR and risk for adverse pregnancy outcomes differ between people with PCD—depending on genotypes—and close monitoring and support might be needed from fertility specialists to increase chances of successful conception.

## Supplementary Material

hoae039_Supplementary_Data

## Data Availability

*Living with PCD* data are available upon reasonable request by contacting C.E.K. (claudia.kuehni@unibe.ch).
