# Supplementary material for: Infertility and pregnancy outcomes among adults with primary ciliary dyskinesia
Source: Hum Reprod Open. 2024 Jun 18;2024(3):hoae039. doi: 10.1093/hropen/hoae039 (PMC11219480; doi:10.1093/hropen/hoae039)
Supplement: hoae039_Supplementary_Data [file hoae039_supplementary_data.docx]

**Supplementary Table S1:** Formulation of relevant questions from the English adult baseline questionnaire and the English female and male fertility questionnaire of the *Living with PCD** study.

| **Question** | | **Answer category** |
| --- | --- | --- |
| **Baseline Questionnaire** | | |
| What is your sex? | Male; Female; Other | |
| Which year were you born? | *text (integer)* | |
| Which country do you live in? | List of all countries worldwide | |
| Which other country? | *text* | |
| What is your height in centimeter? If you would rather enter feet and inches, please go to the next question | *text (integer)* | |
| What is your height in feet and inches? | *text* | |
| What is your weight in kilogram? If you would rather enter weight in pounds, please go to the next question | *text (integer)* | |
| What is your weight in pounds? | *text (integer)* | |
| Do you smoke? | No, I have never smoked; Yes, rarely; Yes, daily; Ex-smoker, I don't smoke anymore | |
| Are any of your organs in a different position compared to most people? (E.g. the heart on the right side instead of on the left) | No; Yes; I don't know | |
| Which year were you diagnosed with PCD? | *text (integer)* | |
| How old were you, when you were diagnosed with PCD? | *text (integer)* | |
| Have you had diagnostic tests for PCD? | No; Yes | |
| Have you had a genetic test (looking for genes that cause PCD)? | No; Yes; I don’t know/I cannot remember | |
| Were any genes found that cause PCD? | No; Yes; I don't know/ I cannot remember/ waiting for results | |
| Which gene was found? | List of all known PCD genes (in 2020) | |
| Which gene was found? (If a second gene was found) | List of all known PCD genes (in 2020) | |
| **Fertility questionnaire** | | |
| Have you ever seen a fertility expert (= a doctor who is specialized in fertility)? | No;  No, but I have an appointment / I am waiting to be referred;  No, but I would like to;  Yes | |
| How old were you when you first saw a fertility expert? If you cannot remember the exact age, please give an approximate age. | *text (integer)* | |
| Who referred you to the fertility expert? | I was referred by my PCD physician.;  I was referred by a doctor other than my PCD physician (e.g. my general practitioner).;  I had to ask for a referral.;  I had to organize it myself.;  Other | |
| Please specify who referred you to the fertility expert | *text* | |
| Have you ever been told by a health care professional that you may have problems with your fertility or that you are infertile? | No; Yes; I don’t know | |
| What reasons for your fertility problems were mentioned? (Tick all that apply) | Reason related to PCD;  Reason related to other causes;  Unknown cause of fertility problems at that time, PCD only diagnosed after;  Unknown reason; I don't know | |
| Please specify what reason for your fertility problems was mentioned | *text* | |
| [only for females] How regular is/was your period during your reproductive years between 18 and 40 years (when you are/were not using hormonal contraceptives, e.g. the pill)? | My period is/was usually regular, occurring every 25-35 days.; My period is/was sometimes regular, sometimes irregular, but my menstrual cycle is only a few days longer or shorter than 25-35 days.;  My period is/was totally irregular or absent.;  Other;  I don't know / I don't remember | |
| [only for females] Please specify how your period is/was during your reproductive years | *text* | |
| [only for females] Have you been diagnosed with any of the following conditions that affect fertility which are not known to be related to PCD? (Tick all that apply) | No;  Polycystic ovaries (many fluid-filled sacs in the ovaries);  Endometriosis (condition where tissue similar to the tissue in the uterus starts to grow in other places of the body); Other problems | |
| [only for females] Please specify which condition you have been diagnosed with | *text* | |
| Have you ever had any tests to check your fertility? | No; Yes | |
| What tests did you have to check your fertility? | [only for females] Checking the flow through the uterine tubes (tubal patency testing, HyCoSy). A liquid is injected into the uterus and then checked by ultrasound or radiography to see if it reaches the abdomen.;  Measurement of fertility hormones;  Other | |
| Please specify what other tests were performed | *text* | |
| How old were you when the tests were performed? If you cannot remember the exact age, please give an approximate age. | *text (integer)* | |
| [only for females] What was the result of the tubal patency testing? | Both tubes were open/permeable.;  One tube was blocked.;  Both tubes were blocked.;  I don't know | |
| Were the levels of fertility hormones normal? | No; Yes; I don’t know | |
| [only for females] Did you have to take medication or have you had any surgical procedures on your ovaries or uterus? | No;  Yes, I had to take medication to have regular ovulation.;  Yes, I had surgical procedures on my ovaries or uterus.;  Yes, other | |
| [only for females] Please specify what treatment or surgical procedure you had | *text* | |
| *For the following questions, only the formulation for females is reported* | | |
| Have you ever tried to become pregnant? | No;  Yes, but it did not work / we are still trying;  Yes, it worked | |
| Have you ever been pregnant? This also includes pregnancies that were terminated or resulted in miscarriages. | No; Yes, once; Yes, twice; Yes, three times; Yes, four times; Yes, five times; Yes, more than five times | |
| How many times have you been pregnant? This also includes pregnancies that were terminated or resulted in miscarriages. | Once; Twice; Three times; Four times; Five times; More than five times | |
| How old were you when you were pregnant the first time? | text (integer) | |
| Was the first/second/third/fourth/fifth/sixth pregnancy a natural pregnancy or a pregnancy as a result of fertility treatment? | Natural pregnancy; Pregnancy with help of fertility treatment; I don't know | |
| How long had you been trying to conceive for the first/second/third/fourth/fifth/sixth pregnancy? | < 6 months; 6 to 12 months; 1 to 2 years; More than 2 years; I don't know / I don't remember | |
| What fertility therapy was used for the first/ second/third/fourth/fifth/sixth pregnancy? (Tick all that apply) | I received medication that stimulated my ovaries (ovarian stimulation).;  My partner's semen (sperm) was directly inserted into my uterus (artificial insemination).;  One of my eggs was combined with my partner's sperm outside the body (in vitro fertilization (IVF)).;  My partner's sperm was injected directly into an egg (intracytoplasmic sperm injection (ICSI)).;  We used donated sperm.;  An egg was donated to me (oocyte donation).;  My partner had to undergo fertility treatment.;  Other;  I don't know | |
| Please specify what fertility therapy was used for the first/second/third/fourth/fifth/sixth pregnancy | *text* | |
| What was the outcome of the first/second/third/fourth/fifth/sixth pregnancy? | The baby was born alive.;  The pregnancy was outside my uterus and had to be terminated (ectopic pregnancy).;  The pregnancy was ended with an intervention (abortion).;  The baby was lost during pregnancy or birth (miscarriage or stillbirth).;  Other | |
| Please specify the outcome of the first/second/third/fourth/fifth/sixth pregnancy | *text* | |
| [only for females] Did your lung health get worse during the first pregnancy? (Tick all that apply) | No;  Yes, I had new or worsening symptoms related to PCD.;  Yes, I saw my PCD doctor more frequently.;  Yes, I had to change or increase my medication during pregnancy.;  Yes, I was admitted to the hospital for PCD-related issues. | |
| How old were you when you tried getting pregnant the first time? | *text (integer)* | |
| How long have you been trying to get pregnant? | < 6 months; 6 to 12 months; 1 to 2 years; More than 2 years; I don’t know | |
| Do you know if the reasons for not conceiving a child are related to your fertility problems or your partner's? (Tick all that apply) | My fertility problems; My partner's fertility problems; Unknown or unexplained fertility problems; I don't know | |
| Did you use any fertility therapy? | *No; Yes* | |
| Why did you not use any fertility therapy? (Tick all that apply) | It was not available where I lived.; It was not covered by the health insurance.; I could not afford it.; I did not want to.; Other; I don't know | |
| Please specify the reason for not using fertility therapy | *text* | |
| What fertility therapy was used? (Tick all that apply) | I received medication that stimulated my ovaries (ovarian stimulation).;  My partner's semen (sperm) was directly inserted into my uterus (artificial insemination).;  One of my eggs was combined with my partner's sperm outside the body (in vitro fertilization (IVF)).;  My partner's sperm was injected directly into an egg (intracytoplasmic sperm injection (ICSI)).;  We used donated sperm.;  An egg was donated to me (oocyte donation).;  My partner had to undergo fertility treatment.;  Other;  I don't know | |
| Please specify what fertility therapy was used | *text* | |

**Living with PCD* is an international, online, participatory study that collects information by questionnaire directly from people with PCD.

PCD, primary ciliary dyskinesia.

**Supplementary** **Table S2:** Definition of abnormal fertility tests and other factors associated with fertility assessed in the *Living with PCD* study*.

| **Test/Condition** | **Definition** | **Sex** |
| --- | --- | --- |
| **BMI > 30/≤ 18 kg/m^2^** | Adults who reported a BMI > 30 or ≤ 18 in the baseline questionnaire.  To calculate BMI, we used self-reported height and weight data from the *Living with PCD* baseline questionnaire. We calculated BMI by dividing weight in kilograms by height in meters squared (kg/m^2^). | Both |
| **Ever smoked/smoking** | Adults who reported that they are currently smoking or that they are ex-smokers in the baseline questionnaire. | Both |
| **Abnormal levels of fertility hormones** | Adults who reported to have abnormal levels of fertility hormones. | Both |
| **Other condition** | Adults who reported having any other condition that could affect fertility in the fertility questionnaire:  - for women: removal of an ovary, low egg count and quality, ovarian dermoid cysts, borderline tumors of the ovary, uterine fibroids, malformation of the uterus, papillomavirus infection.  - for men: varicocele. | Both |
| **Period totally irregular or absent** | Women who reported that their period was totally irregular or absent. | Women |
| **Endometriosis** | Women who reported suffering from endometriosis. | Women |
| **Polycystic ovaries** | Women who reported suffering from polycystic ovaries. | Women |
| **Problems with tubal patency** | Women who reported that either one or both fallopian tubes were blocked. | Women |
| **Abnormal ultrasound of testicles** | Men who reported abnormal ultrasound of testicles. | Men |
| **Abnormal result of sperm analysis** | Men who reported an abnormal sperm analysis such as low sperm concentration, low sperm mobility, low sperm vitality, too few sperm with normal shape or other reasons for abnormal sperm result. | Men |

PCD, primary ciliary dyskinesia.

**Living with PCD* is an international, online, participatory study that collects information by questionnaire directly from people with PCD.

**Supplementary** **Table S3:** Comparison of basic characteristics of adults participating in *Living with PCD** who did or did not complete the questionnaire on fertility (N = 482).

|  |  | **Completed questionnaire** | **Did not complete questionnaire** |
| --- | --- | --- | --- |
|  |  | **n (%)** | **n (%)** |
|  |  | **n = 265** | **n = 217** |
| **Age at survey** | Median (IQR) | 44 (33-54) | 36 (26-47) |
|  | 18-30 y | 47 (37) | 80 (63) |
|  | 31-45 y | 100 (56) | 78 (44) |
|  | > 45 y | 118 (67) | 59 (33) |
|  |  |  |  |
|  |  |  |  |
| **Sex** | Women | 180 (56) | 142 (44) |
|  | Men | 85 (54) | 73 (46) |
|  | Other | 0 (0) | 2 (100) |
|  |  |  |  |
|  |  |  |  |
| **Countries^a^** | USA | 41 (47) | 47 (53) |
|  | England | 39 (46) | 45 (54) |
|  | Germany | 37 (65) | 20 (35) |
|  | Switzerland | 26 (76) | 8 (24) |
|  | Italy | 19 (50) | 19 (50) |
|  | France | 16 (59) | 11 (41) |
|  | Australia | 12 (71) | 5 (29) |
|  | Canada | 10 (71) | 4 (29) |
|  | Netherlands | 9 (60) | 6 (40) |
|  | Scotland | 7 (78) | 2 (22) |
|  | Norway | 6 (67) | 3 (33) |
|  | Denmark | 5 (56) | 4 (44) |
|  | Spain | 4 (44) | 5 (56) |
|  | Sweden | 3 (43) | 4 (57) |
|  | Austria | 3 (50) | 3 (50) |
|  | Ireland | 3 (60) | 2 (40) |
|  | other European countries | 17 (65) | 9 (35) |
|  | other countries | 8 (29) | 20 (71) |

**Living with PCD* is an international, online, participatory study that collects information by questionnaire directly from people with PCD.

IQR, interquartile range. PCD, primary ciliary dyskinesia. y, years. All characteristics are presented as n and row % or as median and interquartile range. ^a^Countries with N≥5 adult participants displayed in table, countries with N<5 were categorised into other European countries and other countries.

**Supplementary Table S4:** Information about fertility characteristics of men participating in *Living with PCD** who completed the fertility questionnaire, by fertility status (N = 85).

|  | | **All men** | | **Fertile** | **Infertile** | **Unknown fertility status^a^** |
| --- | --- | --- | --- | --- | --- | --- |
|  | **n (%)** | | **n (%)** | | **n (%)** | **n (%)** |
|  | **N = 85** | | **n = 11** | | **n = 39** | **n = 35** |
| **Age at first pregnancy attempt** | **n = 50** | | **n = 11** | | **n = 39** | **NA** |
| Median (IQR; range) | 31 (28-35; 19-45) | | 29 (28-32, 19-38) | | 31 (28-35; 20-45) | NA |
| 18-30 y | 23 (46) | | 8 (73) | | 15 (38) | NA |
| 31-45 y | 25 (50) | | 3 (27) | | 22 (56) | NA |
| Missing | 2 (4) | | 0 (0) | | 2 (5) | NA |
|  |  | |  | |  |  |
| **Sperm analysis done** | n = 62 | | n = 6 | | n = 37 | n = 19 |
| Normal sperm analysis | 3 (5) | | 1 (17) | | 1 (3) | 1 (5) |
| Low sperm concentration | 25 (40) | | 2 (33) | | 14 (38) | 9 (47) |
| Low sperm mobility | 47 (76) | | 2 (33) | | 32 (86) | 13 (68) |
| Low sperm vitality | 7 (11) | | 0 (0) | | 7 (19) | 0 (0) |
| Too few sperm with normal shape | 6 (10) | | 1 (17) | | 3 (8) | 2 (11) |
| Other reasons for abnormal sperm result | 2 (3) | | 1 (17) | | 0 (0) | 1 (5) |
| I don’t know result | 1 (2) | | 0 (0) | | 0 (0) | 1 (5) |
|  |  | |  | |  |  |
| **Conditions that affect fertility** | **17 (20)** | | **0 (0)** | | **12 (31)** | **5 (14)** |
| - Ever smoked/smoking | 14 (16) | | 0 (0) | | 9 (23) | 5 (14) |
| - Abnormal ultrasound of testicles | 3 (4) | | 0 (0) | | 2 (5) | 1 (3) |
| - Abnormal levels of fertility hormones | 3 (4) | | 0 (0) | | 3 (8) | 0 (0) |
| - Other condition | 1 (1) | | 0 (0) | | 1 (3) | 0 (0) |

**Living with PCD* is an international, online, participatory study that collects information by questionnaire directly from people with PCD.

IQR, interquartile range. NA, not applicable. PCD, primary ciliary dyskinesia. y, years. All characteristics are presented as n and column % unless otherwise stated. ^a^We classified people as unknown fertility status if they reported not having tried to conceive or if they tried for less than 12 months.

**Supplementary Table S5:** Information about fertility characteristics of women participating in *Living with PCD** who completed the fertility questionnaire, by fertility status (N = 180).

|  | **All women** | **Fertile** | **Infertile** | **Unknown fertility status^a^** |
| --- | --- | --- | --- | --- |
|  | **n (%)** | **n (%)** | **n (%)** | **n (%)** |
|  | **N = 180** | **n = 46** | **n = 72** | **n = 62** |
| **Age at first pregnancy attempt** | **n = 118** | **n = 46** | **n = 72** | **NA** |
| Median (IQR; range) | 30 (27-32; 16-40) | 28 (24-33; 16-39) | 30 (27-32; 19-40) | NA |
| 16-30 y | 75 (64) | 32 (70) | 43 (60) | NA |
| 31-45 y | 42 (36) | 14 (30) | 28 (39) | NA |
| Missing | 1 (1) | 0 (0) | 1 (1) | NA |
|  |  |  |  |  |
| **Other conditions associated with infertility** | **78 (43)** | **22 (48)** | **36 (50)** | **20 (32)** |
| - BMI > 30, ≤ 18 | 25 (14) | 9 (20) | 12 (17) | 4 (6) |
| - Ever smoked/smoking | 16 (9) | 4 (9) | 4 (6) | 8 (13) |
| - Period totally irregular/absent | 11 (6) | 2 (4) | 5 (7) | 4 (6) |
| - Endometriosis | 16 (9) | 3 (7) | 10 (14) | 3 (5) |
| - Polycystic ovaries | 12 (7) | 2 (4) | 8 (11) | 2 (3) |
| - Problems with tubal patency | 7 (4) | 2 (4) | 5 (7) | 0 (0) |
| - Abnormal levels of fertility hormones | 6 (3) | 1 (2) | 5 (7) | 0 (0) |
| - Other condition | 9 (5) | 2 (4) | 5 (7) | 2 (3) |

**Living with PCD* is an international, online, participatory study that collects information by questionnaire directly from people with PCD.

IQR, interquartile range. NA, not applicable. PCD, primary ciliary dyskinesia. y, years. All characteristics are presented as n and column % unless otherwise stated. ^a^We classified people as unknown fertility status if they reported not having tried to conceive or if they tried for less than 12 months.

**Supplementary Table S6:** Characteristics of women participating in *Living with PCD** who completed the fertility questionnaire and reported an ectopic pregnancy (n = 9).

|  | #1 | #2 | #3 | #4 | #5 | #6 | #7 | #8 | #9 |
| --- | --- | --- | --- | --- | --- | --- | --- | --- | --- |
|  |  |  |  |  |  |  |  |  |  |
| Age at first ectopic pregnancy (in y)^a^ | 21-25^b^ | 21-25^b^ | 36-40 | 21-25 | 26-30 | 16-20 | 31-35 | 31-35 | 26-30^b^ |
| BMI (kg/m^2^) | 28 | 34 | 29 | 34 | 30 | 27 | 19 | 24 | 18 |
| Number of ectopic pregnancies | 2 | 3 | 1 | 1 | 2 | 2 | 1 | 2 | 1 |
| Mode of fertilization for ectopic pregnancies | both natural pregnancies | 2 natural pregnancies, 1 NA | natural pregnancy | natural pregnancy | both natural pregnancies | both natural pregnancies | IVF | both natural pregnancies | IVF |
| Pregnancy rank of ectopic pregnancy | first, second | first, second, fifth | third | first | first, second | first, second | first | second, third | second |
| Miscarriage before ectopic pregnancy | No | No | Yes | No | No | No | No | No | Yes |
| Number of live births (mode of fertilization) | 3 (all with ICSI) | 2 (both with IVF) | 1 (natural pregnancy) | 1 (natural pregnancy) | 1 (natural pregnancy) | 0 | 2 (both with IVF) | 2 (1 natural pregnancy,  1 with IVF) | 0 |
| Ever smoked/smoking | No | No | No | No | No | Yes | No | No | No |
| Tubal surgery | No | No | No | Ablation | No | No | No | No | No |
| Tubal patency | Both open | Not tested | Both open | Not tested | Not tested | Not tested | Not tested | Not tested | Not tested |
| Endometriosis | No | No | No | No | No | No | No | No | Yes |
| Situs inversus | No | No | Yes | Yes | Yes | Yes | Yes | No | Yes |
| Gene | NA | NA | NA | DNAH5 | DNAH5 | NA | NA | NA | NA |

**Living with PCD* is an international, online, participatory study that collects information by questionnaire directly from people with PCD.

MAR, medically assisted reproduction. NA, not available. PCD, primary ciliary dyskinesia. y, years. ^a^To ensure anonymity, we only provide age groups. ^b^Age at ectopic pregnancy was not reported. We used age at first pregnancy attempt (for pregnancies without MAR) or age at fertility specialist visit (for pregnancies with the use of MAR) as a proxy.
